# Global Analysis of Circulating Immune Cells by Matrix-Assisted Laser Desorption Ionization Time-of-Flight Mass Spectrometry

**DOI:** 10.1371/journal.pone.0013691

**Published:** 2010-10-27

**Authors:** Richard Ouedraogo, Christophe Flaudrops, Amira Ben Amara, Christian Capo, Didier Raoult, Jean-Louis Mege

**Affiliations:** Unité de Recherche sur les Maladies Infectieuses Tropicales et Emergentes, Centre National de la Recherche Scientifique - Institut de Recherche pour le Développement Unité Mixte de Recherche 6236, Université de la Méditerranée, Faculté de Médecine, Marseille, France; BMSI-A*STAR, Singapore

## Abstract

**Background:**

MALDI-TOF mass spectrometry is currently used in microbiological diagnosis to characterize bacterial populations. Our aim was to determine whether this technique could be applied to intact eukaryotic cells, and in particular, to cells involved in the immune response.

**Methodology/Principal Findings:**

A comparison of frozen monocytes, T lymphocytes and polymorphonuclear leukocytes revealed specific peak profiles. We also found that twenty cell types had specific profiles, permitting the establishment of a cell database. The circulating immune cells, namely monocytes, T lymphocytes and polymorphonuclear cells, were distinct from tissue immune cells such as monocyte-derived macrophages and dendritic cells. In addition, MALDI-TOF mass spectrometry was valuable to easily identify the signatures of monocytes and T lymphocytes in peripheral mononuclear cells.

**Conclusions/Significance:**

This method was rapid and easy to perform, and unlike flow cytometry, it did not require any additional components such as specific antibodies. The MALDI-TOF mass spectrometry approach could be extended to analyze the cell composition of tissues and the activation state of immune cells.

## Introduction

Immune cells are characterized by specific morphologies and functions, which can be used to identify different immune cell types. This is illustrated by the use of flow cytometry to identify immune cell populations based on the recognition of increasing numbers of membrane antigens by specific antibodies. This method has been widely applied in the fields of immunology and hematology. The development of systems biology approaches (such as transcriptomics) has enabled cell subsets to be identified through their characteristic transcriptional signatures. For example, it has been recently reported that circulating lymphocytes and polymorphonuclear cells (PMNs) exhibit gene expression signatures reflecting the enrichment of genes encoding specific surface proteins that can be used as biomarkers for estimating the abundance of these cell types within complex tissues [1]. This approach enables discrimination between cells in the same lineage but at different stages and between cells that have differentiated, such as the differentiation of human monocytes into macrophages or dendritic cells (DCs) [2]. However, changes in mRNA levels do not necessarily reflect the altered expression of proteins [3]. A proteomic approach that analyzes signatures based on protein expression would provide a robust method with power similar to that of the transcriptomic approach.

Mass spectrometry (MS) is a key tool in cell proteomics [4]–[6]. This technique, based on mass determination [7], is currently used to identify proteins, their amino-acid sequences and their post-translational modifications [8], [9]. This method can also be used for the identification and sequencing of DNA, RNA and sugars [9], [10]. MALDI-TOF (matrix-associated laser desorption ionization/time of flight) MS is used to identify unknown protein or peptide sequences in fractionated cells [9]. Coupled with two-dimensional gels, MALDI-TOF MS can be used to create proteomic maps of cell types such as macrophages [4] and of intracellular compartments [11]. MALDI-TOF MS has been recently introduced into microbiology laboratories to identify [12], [13] and classify bacterial species using intact bacteria [14], [15]. In 2008 a large number of bacterial species present in clinical specimens were identified using databases established from isolated species [16], [17]. In 2006, MALDI-TOF MS has been applied to mammalian cells from three cell lines after lysis in 2,5-dihydroxybenzoic acid matrix solution. In these conditions, it has been possible to discriminate the different mammalian lines [18]. Recently, MALDI-TOF MS has been applied to eukaryotic cell lines to provide rapid characterization of cultured cells. However, the method used to analyze these cultured cells involved two steps of ethanol inactivation and formic acid/acetonitrile extraction [19]. To our knowledge, MALDI-TOF MS has not yet been directly applied to intact eukaryotic cells.

Our objective was to determine whether intact immune cells exhibited reproducible and specific signatures in MALDI-TOF MS. We found that this approach was useful for discriminating between immune cells. For example, circulating T lymphocytes, monocytes and PMNs as well as monocyte-derived macrophages and DCs all exhibited distinct spectra. We describe the first elements of a database that will be useful for studying cell subsets in tissues and possibly their activation state.

## Methods

### Ethics Statement

Healthy human placentas were collected after informed and written consent obtained from each subject, and the study was approved by the Ethics Committee of the Université de la Méditerranée, Marseille, France.

### Human primary cells

Peripheral blood mononuclear cells (PBMCs) from healthy donors were isolated from leukopacks (Etablissement Français du Sang) by Ficoll gradient (MSL, Eurobio) and suspended in RPMI 1640 containing 20 mM HEPES (Invitrogen), as previously described [20]. Monocytes and T lymphocytes were isolated using CD14 and CD3 MicroBeads, respectively, and the MACS separation system (Miltenyi Biotec) according to the manufacturer's protocol. Monocytes were cultured for seven days in RPMI 1640 containing 10% human AB serum, 2 mM L-glutamine, 100 UI/mL penicillin and 100 µg/mL streptomycin to obtain monocyte-derived macrophages (MDMs), as previously described [21]. More than 90% of cells were macrophages as assessed by flow cytometry using CD68 as a specific marker. To obtain dendritic cells (DCs), monocytes were treated with 1,000 U/ml of human recombinant granulocyte macrophage-colony stimulating factor (Peprotech Inc.) and 500 U/ml of human recombinant interleukin 4 (Tebu-Bio) in RPMI 1640 containing 10% fetal calf serum (FCS), L-glutamine and antibiotics for seven days. The cells obtained expressed high levels of CD11c and CD1a, and low levels of CD14 and CD68. PMNs obtained after Ficoll gradient were prepared by sedimentation of red blood cells (RBCs) with 1.5% (w/v) dextran T500 (Pharmacosmos), as previously described [22]. Cell supernatants were centrifuged at 700× *g*, and a hypotonic shock of 30 s was applied to cell pellets to remove contaminating RBCs; more than 98% of the cells were PMNs. RBCs were diluted and suspended in RPMI 1640 containing 20 mM HEPES before use.

Human placentas were collected, and small pieces were rinsed several times and digested in Hank's balanced salt solution containing DNase I (Sigma-Aldrich, 300 units/mL) and 2.5% trypsin (Invitrogen) for 2×45 min, as previously described [23]. Isolated cells were filtered and centrifuged at 1,200× *g* on 25–60% Percoll gradients (GE Healthcare). Cells present at the interface were subjected to positive selection using MicroBeads coupled with rat anti-mouse IgG2 antibodies (Miltenyi Biotec) and mouse antibodies directed against epidermal growth factor receptor (Santa Cruz Biotechnology), a specific marker of trophoblasts. Primary trophoblasts were cultured in Dulbecco's Minimum Eagle's Medium (DMEM)-F12-Ham containing 10% FCS and antibiotics.

### Mouse primary cells

Bone marrow-derived macrophages (BMDMs) were generated from six- to eight-week-old C57BL/6 mice killed by cervical dislocation, as previously described [24]. In brief, the bone marrow was flushed out from femurs and tibias in DMEM supplemented with 10% FCS, 2 mM L-glutamine and antibiotics. Cells were cultured in DMEM supplemented with 10% FCS, L-glutamine, antibiotics and 15% L929 cell supernatant rich in granulocyte macrophage-colony stimulating factor for seven days.

### Cell lines

The human monocytic leukemia cell line THP1 (ATCC N° TIB-202) and the murine J774 (ATCC N° TIB-67) and canine DH82 (ATCC N° CRL-10389) macrophage cell lines were cultured in RPMI 1640 containing 10% FCS, L-glutamine and antibiotics. The human T cell leukemia cell line C8166 that stably expresses the CCR5 chemokine receptor [25] was kindly provided by Dr. G. Querat (Marseille). Murine L929 (ATCC N° CCL-1) is a fibroblastic-like cell line. The epithelial cells used were human HeLa (ATCC N° CCL-2) cells and 293T cells (ATCC N° CRL-1573). Fibroblast-like cells and epithelial cells were cultured in DMEM containing 10% FCS, L-glutamine and antibiotics. The human BeWo and JEG trophoblast cell lines were obtained from ATCC (N° CCL-98 and HTB-36, respectively) and were cultured in DMEM F-12 Ham medium in the same way as primary trophoblasts (Invitrogen). Confluent monolayers were trypsinized twice a week and used for a maximum of five passages.

### Non-mammalian cells

The XTC-2 cell line, derived from *Xenopus laevis*, was cultured in Leibowitz-15 medium containing L-glutamine, amino-acids, 5% FCS and 2% tryptose phosphate (Invitrogen) at 28°C, as previously described [26]. Amoebae including *Acanthamoeba polyphaga* (ATCC N° 30461), *Acanthamoeba castellanii* (ATCC N° 30234), *Hartmannella vermiformis* (ATCC N° 50237) and *Poteriochromonas melhamensis* (ATCC N° 11532) were grown in peptone yeast-extract glucose (PYG) medium consisting of 20 g/L roteosepeptone, 1 g/L yeast extract, 1 g/L sodium citrate, 4 µM MgSO_4_, 0.4 µM CaCl_2_, 2.5 µM Na_2_HPO_4_, 2.5 µM KH_2_PO_4_, 5 µM (NH_4_)_2_FeII(SO_4_)_2_, and 0.1 M glucose for three days at 32°C, as previously described [27]. Harvested amoebae were washed twice in Page's amoeba saline (PAS) to remove most nutrients and diluted in sterile PBS.

### MALDI-TOF MS

Primary cells and cell lines were obtained from at least ten different isolation procedures, and MALDI-TOF MS was performed at least in duplicate on each cell isolate. Isolated cells (10^6^ cells per assay) were centrifuged at 300× *g* for 5 min, washed in sterile PBS without Ca^2+^ or Mg^2+^, and again centrifuged to remove medium traces. Cell pellets were collected in 10 µL of sterile PBS without Ca^2+^ or Mg^2+^ and were frozen at −80°C for 2–3 days before analysis. When monocytes (10^6^ cells) and T lymphocytes (10^6^ cells) were mixed, cell pellets were collected in 20 µL of PBS. In some experiments, human monocytes were lysed with a RIPA buffer containing 25 mM Tris, 750 mM NaCl, 5% TritonX-100 (Sigma-Aldrich), 25 mM MgCl_2_, 5 mM EDTA and 0.5% sodium dodecyl sulfate, or they were sonicated in the presence of complete protease inhibitor cocktail tablets (Roche Applied Science). MALDI-TOF MS was performed using an AutoflexII mass spectrometer and FlexControl software (Bruker Daltonics), as previously described [13], [28]. In brief, after samples were thawed, 1 µl was deposited on the MALDI target in which 1 µl of acid-α-cyano-4 hydroxy-cynnamic (HCCA) matrix was added. This matrix consisted of a 10 mg/mL solution of HCCA diluted in 50% acetonitrile and 25% Milli-Q grade water containing 10% trifluoroacetic acid. The evaporation that gradually took place at room temperature allowed the formation of HCCA crystals containing the dispersed samples. The crystals were illuminated with a nitrogen laser (337 nm, 3 ns pulse width), and released ions were extracted with an accelerating voltage of 20 kV in linear mode, and extraction delay times varied from 280 to 320 ns depending on the chosen mass range (*m/z* range of 2000–20,000). Each spectrum resulted from the sum of positive ions obtained after 525 laser shots performed in seven different regions of the analyzed sample. A signal-to-noise ratio of 3 was selected to define peaks, with a maximum of 100 peaks per spectrum.

### Spectrum analysis and database acquisition

The ClinProTools version 2.2 software (Bruker Daltonics) was used to analyze the variability between samples from different blood donors. The Gel View representation displays two types of spectra arranged in a pseudo-gel format. The 2D Peak Distribution View automatically selects two peaks, and their relative intensities are expressed as a 2D representation. The Biotyper version 2.0 (Bruker Daltonics) software was used to create an averaged spectrum for each cell type corresponding to at least 20 individual spectra obtained from at least ten different cell cultures tested in duplicate. Baselines were automatically subtracted from spectra, and the background noise was smoothed during acquisition through the FlexControl software. This reference was validated by other samples from the same cell type. The Biotyper software realigns acquired spectra from each cell type and automatically creates an average spectrum using default Biotyper software settings provided by the manufacturer. These settings were the same than those used in routine bacteriology [13]. Briefly, the sensitivity or the maximum tolerated error on the values of mass spectra and spectrum shift was 8000 particles per million. The minimum frequency to benchmark selection of peaks was 25%, and only peaks with a signal/noise intensity above background were selected by the software. The cell-type reference consisting of 70 peaks was added to the database. The Biotyper software was also used to identify unknown spectra by comparison with reference spectra, as recently described for the identification and classification of microorganisms [13]. We used the score values proposed by the manufacturer for microorganisms: values between 0.000 and 1.699 did not allow reliable cell identification; values between 1.700 and 1.999 allowed probable cell identification; scores higher than 2.0 were considered statistically significant and allowed the confident identification of different cell species. Finally, we used Multiexperiment Viewer (MeV) version 4.3 software (http://www.tm4.org/) to perform hierarchical clustering with dendrogram representations of collected MALDI-TOF MS data. The mass values of peaks (with an area equal or greater than to 20) of the reference spectrum of each cell type were selected after treatment of these spectra (smoothing and subtraction of background noise) by the FlexAnalysis software. For a given spectrum, only the weight values with a gap strictly greater than 3 were selected; for spectra of different cell types, the gap must be greater than or equal to 3. The *m/z* values of peaks from given spectra were transferred into an Excel file with a value of +1. The value of −1 was assigned to *m/z* positions without a peak, and conventional color code was applied to hierarchical clustering representation.

## Results

### MALDI-TOF MS analysis of monocytes

**Figure 1:**
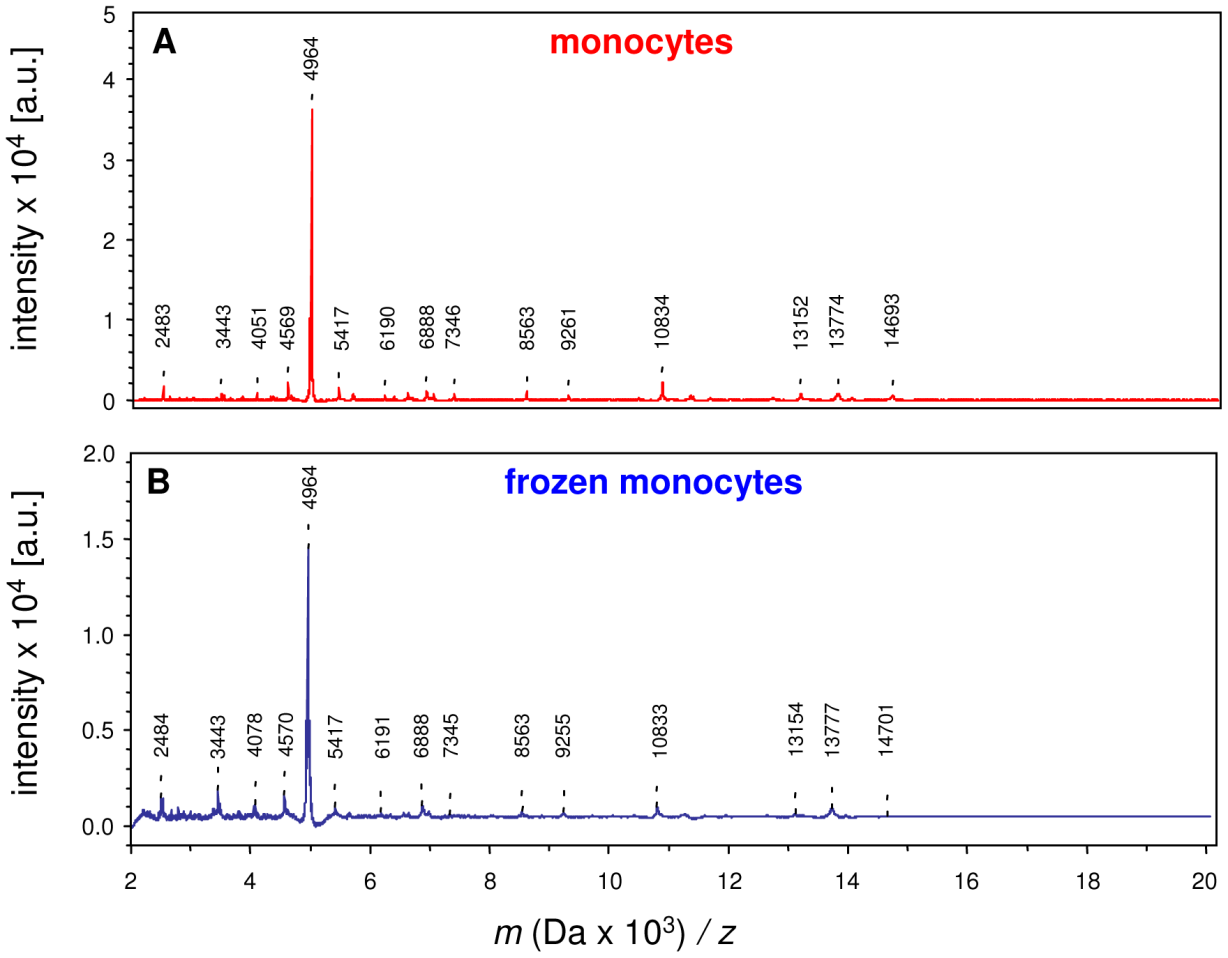
MALDI-TOF MS spectra of monocytes. Human monocytes (10^6^ cells per assay) were collected in 10 µl of PBS, and 1 µl was deposited on the MALDI target. Representative MALDI-TOF MS spectra are shown for A, freshly isolated and B, frozen monocytes.

In the first series of experiments, the MALDI-TOF MS signature of monocytes isolated with CD14 microbeads was analyzed. When 10^6^ monocytes per assay were used, several peaks with different intensities were detected (Fig. 1A), whereas no peak was observed in the control matrix (supplementary Fig. S1A). A major peak was observed at 4964 *m/z*, and several minor peaks were detected between 2,000 and 15,000 *m/z*. Note that the software identified more than 100 peaks per spectrum. In a second series of experiments, monocytes were either frozen at −80°C for two days, lysed using a lysis buffer or sonicated. The freezing procedure did not alter the monocyte signature (Fig. 1B). In contrast, when the monocytes were lysed, diffuse spectra were obtained (supplementary Fig. S1B). When the monocytes were sonicated, the spectra showed peaks that did not correspond to those observed with viable monocytes (supplementary Fig. S1C). Consequently, only frozen samples were used in subsequent experiments.

The effect of monocyte concentration on the presence and positions of peaks was then assessed. Increasing the initial cell concentration (10^6^ cells) by 5- or 10-fold did not modify the position or the intensity of detected peaks, but it did increase the background. Using 10^5^ monocytes was insufficient to detect the full range of peaks that were detected with 5×10^5^ or 10^6^ monocytes. As a consequence, further experiments were performed using 10^6^ frozen cells per assay. Taken together, these results showed that monocyte freezing was a very simple method that permitted delayed handling of samples.

### MS analysis of circulating cells

**Figure 2:**
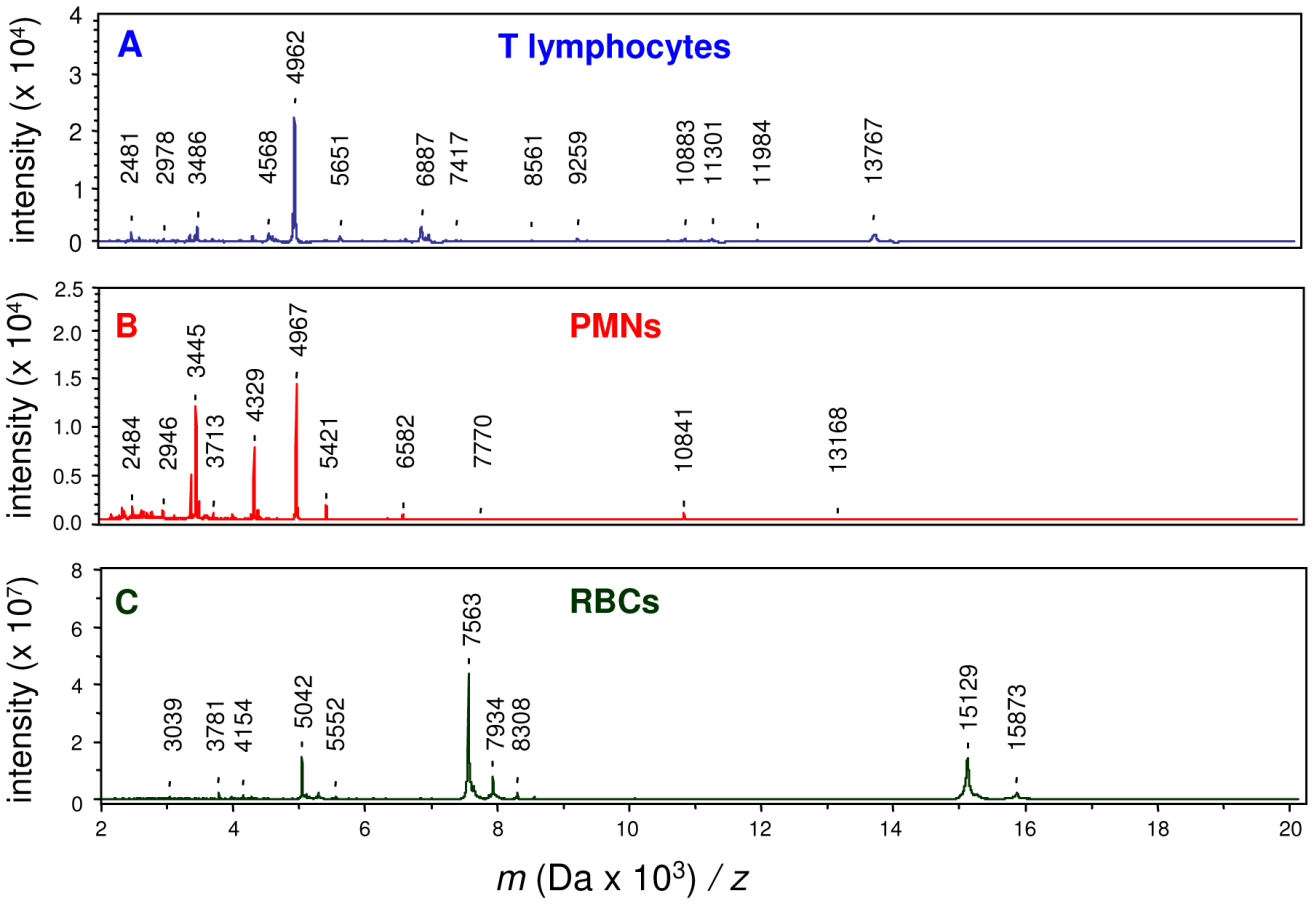
MALDI-TOF MS spectra of circulating cells. T lymphocytes (A), PMNs (B) and RBCs (C) were isolated from a healthy blood donor. Representative MALDI-TOF MS spectra are shown.

**Figure 3:**
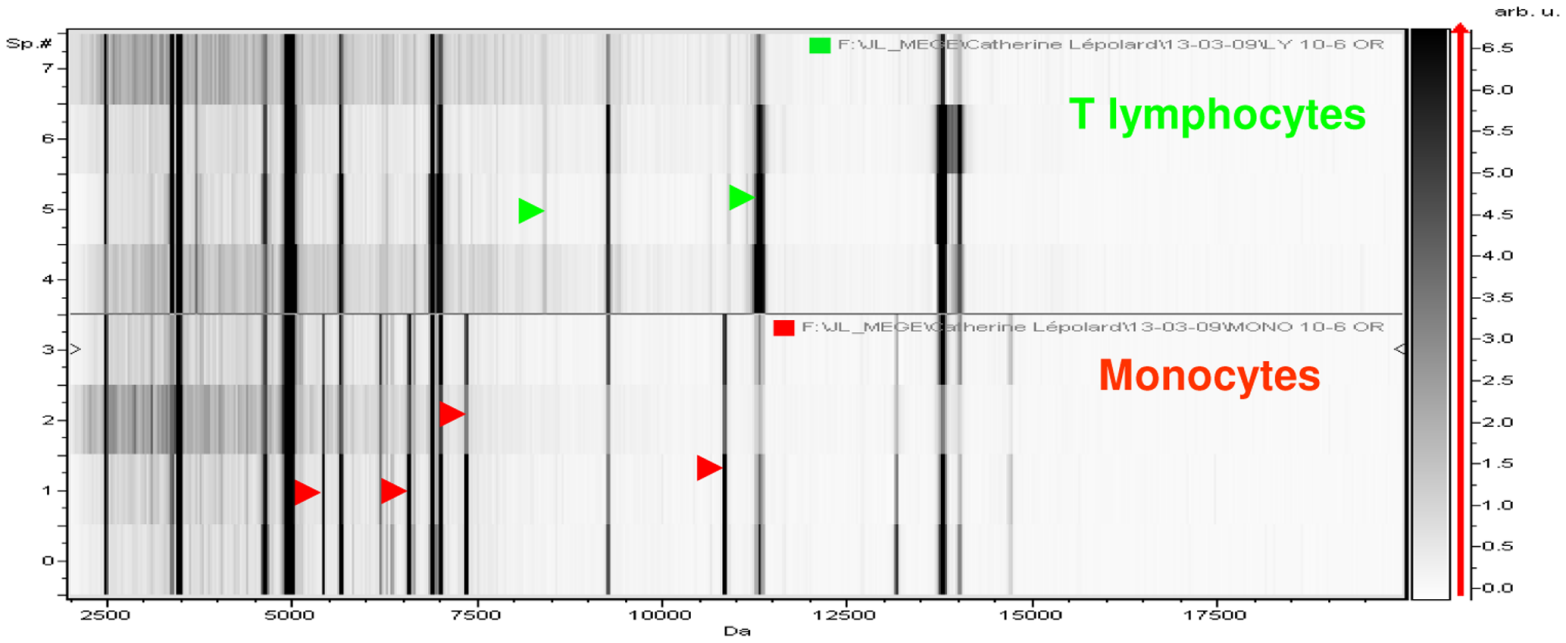
Gel view representation of monocytes and T lymphocytes. Monocytes and T lymphocytes were isolated from a healthy blood donor. MALDI-TOF MS spectra were analyzed using the ClinProTools software, and the spectra are presented in Gel View representation. Representative spectra are shown with *m/z* values on the x-axis and the peak intensity (in arbitrary units) on the y-axis. Major differences between monocytes and T lymphocytes are indicated by arrowheads.

We then compared the profiles of circulating cells isolated from a healthy blood donor. The MALDI-TOF MS signature of T lymphocytes was distinct from that of monocytes (compare Fig. 2A with Fig. 1B). The gel view representation created by the ClinProTools version 2.2 software allowed the comparison of individual spectra (Fig. 3). Peaks at 5418, 6577, 7345 and 10.836 *m/z* were present in monocytes but not in T lymphocytes. Conversely, a peak at 8412 *m/z* present in T lymphocytes was lacking in monocytes. Some peaks were common to both monocytes and T lymphocytes (e.g., the peak at 11360 *m/z*). Similarly, the MALDI-TOF MS spectrum of PMNs (Fig. 2B) differed from those of both monocytes and T lymphocytes. Indeed, the intensity (see the peak at 3445 *m/z*) and presence (see the peak at 4329 *m/z*) of certain peaks distinguished PMNs from both monocytes and T lymphocytes. Finally, the MALDI-TOF MS spectrum of RBCs (Fig. 2C) differed dramatically from those of monocytes, T lymphocytes and PMNs. First, the intensity of the detected peaks was lower by 1000-fold in RBCs. Second, the great majority of peaks (see, for example, the peaks at 5042, 7563, 11129 and 15873 *m/z*) were present only in RBCs, whereas other peaks common to monocytes, T lymphocytes and PMNs, such as the peaks at 2484, 4964 m/z, were lacking in RBCs.

**Figure 4:**
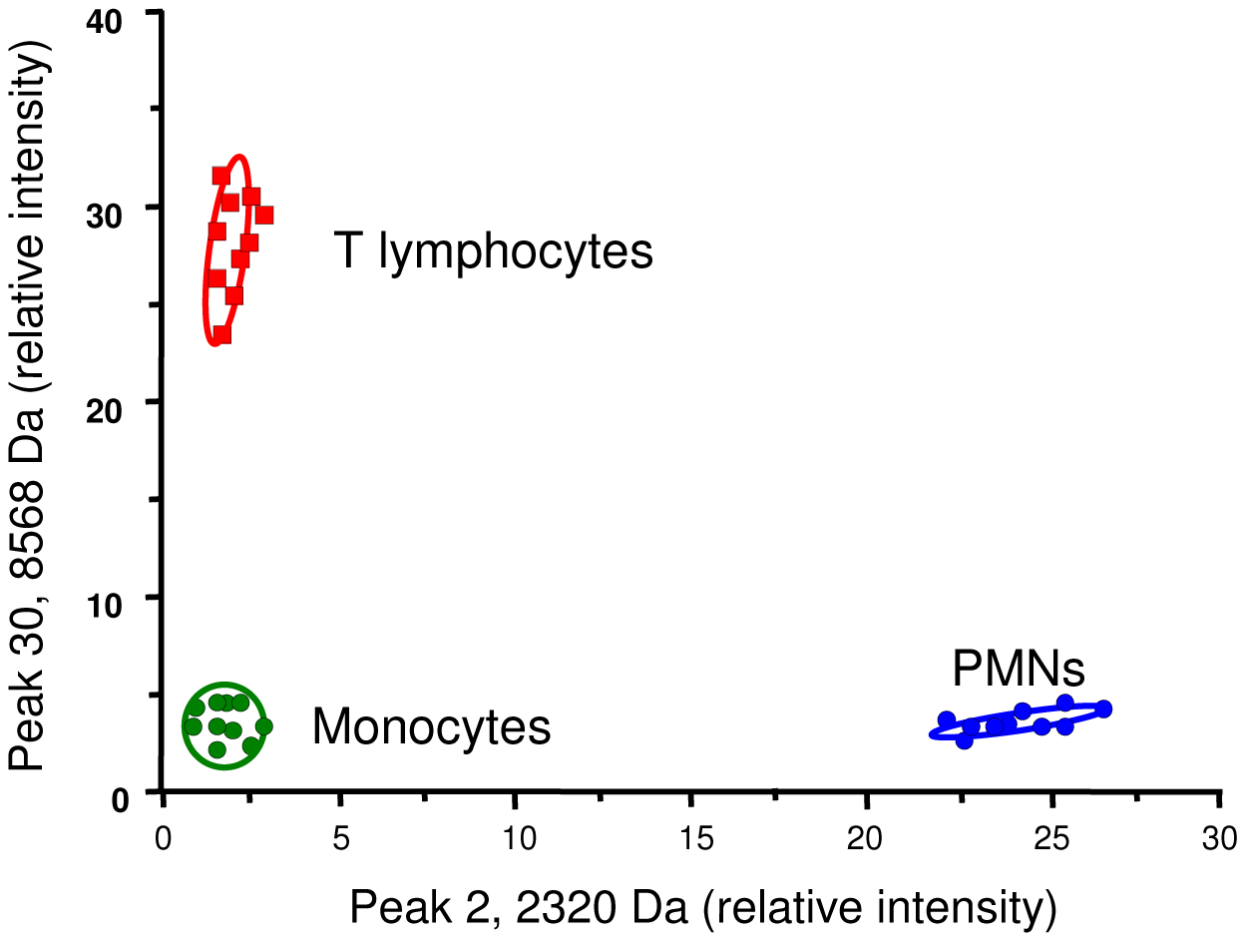
Reproducibility of MALDI-TOF MS signatures. Monocytes (in green), T lymphocytes (in red) and PMNs (in blue) were isolated from ten healthy blood donors. MALDI-TOF MS spectra were analyzed using the ClinProTools software and 2D Peak Distribution View. The relative intensities of the two peaks automatically selected were homogenous among blood donors, and the ellipses represent the standard deviation within each cell population (monocytes, T lymphocytes and PMNs, respectively).

**Figure 5:**
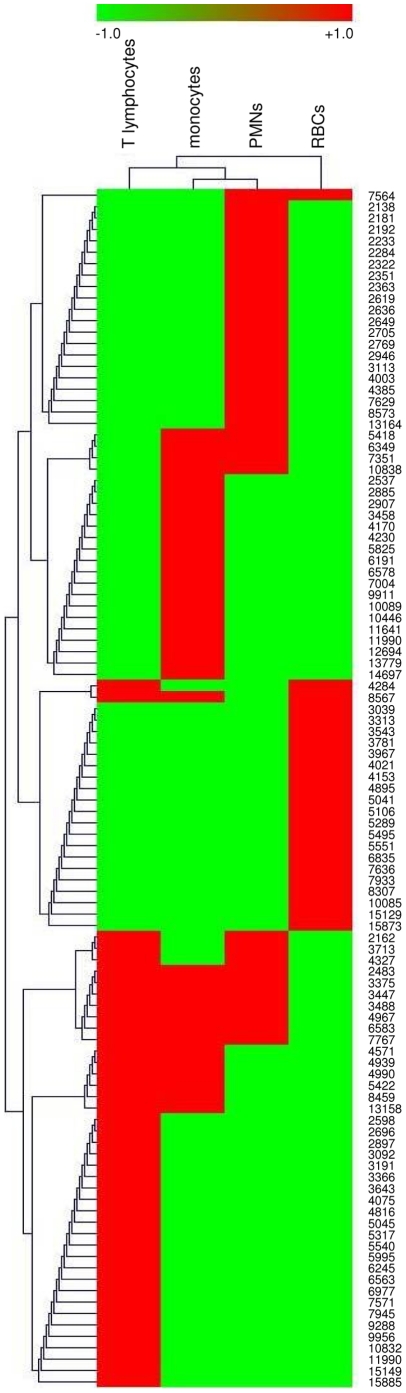
Hierarchical clustering of circulating cells. Monocytes, T lymphocytes, PMNs and RBCs were isolated from a healthy blood donor. MALDI-TOF MS spectra were analyzed using MeV software. A conventional value of +1 was assigned to the m/z values of spectra (in red) and −1 to m/z positions without peaks (in green).

The reproducibility of the signatures of monocytes, T lymphocytes and PMNs was tested using ten different donors. The 2D representation provided by the ClinProTools version 2.2 software illustrates the differences between monocytes, T lymphocytes and PMNs, and it shows that their signatures are remarkably homogenous (Fig. 4). The MALDI-TOF MS data was then clustered hierarchically using the MeV software. The presence of peaks is represented in red (Fig. 5). Clearly, RBCs did not cluster with the other circulating cells. Monocytes clustered with PMNs while T lymphocytes were nearer to monocytes than PMNs. These results suggest that MALDI-TOF MS profiles of circulating cells are reproducible and specific.

### Development of a cell database

Because MALDI-TOF MS profiles seemed to be specific for different types of circulating cells, we created a cell database using the BioTyper version 2.0 software. We included primary myeloid cells such as human MDMs and murine BMDMs in the database. We also included human THP-1 myelomonocytic cells and the murine J774 and canine DH82 macrophage cell lines. Because circulating monocytes differentiate into MDMs or DCs depending on culture conditions, we also assessed the ability of MALDI-TOF MS to discriminate between MDMs, monocyte-derived DCs, and circulating monocytes. Similarly, circulating CD3 T cells were compared to a human CCR5-transfected leukemia cell line. We also analyzed one fibroblast-like cell line, murine L929 cells, and two epithelial cell lines, consisting of human HeLa and 293T cells. Additionally, we compared human placenta trophoblasts with the human BeWo and JEG trophoblast cell lines. Finally, we selected non-mammalian cells such as a *Xenopus* cell line and different types of amoebae for analysis.

**Figure 6:**
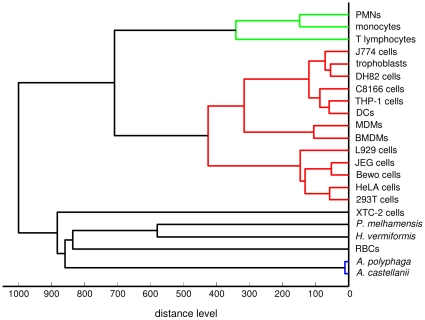
Dendrogram of 22 eukaryotic cell types. MALDI-TOF MS was performed on 22 cell types with at least 20 spectra per cell type. An averaged spectrum for each cell type was added to the database using the BioTyper software and the dendrogram creation method.

The mean spectra of the 22 different cell types were introduced into the database, allowing for an accurate comparison. The cell database was then used to classify and clusterize the various primary cells and cell lines (Fig. 6). Two major clusters were found. The first one contained an invertebrate cell line, several amoebae and (surprisingly) human RBCs. The second cluster included mammalian immune cells and cell types. Among this latter cluster, several branches were identified. The branch that included circulating immune cells could be divided into three specific branches: T lymphocytes, monocytes and PMNs. It is noteworthy that monocyte-derived macrophages and DCs were present in distinct branches, along with several macrophage cell lines (J774, DH82, THP-1) and a T cell line (C8166 cells). Two trophoblast cell lines (i.e., JEG and BeWo cells) clustered together but were distant from primary trophoblasts. Epithelial cells also clustered together (HeLa and 293T cells). Finally, non-immune cell lines including epithelial, fibroblastic and trophoblast cells were located in a branch distinct from immune cells. These results show that MS profiles discriminated immune cells from other eukaryotic cells, and it positioned circulating immune cells (monocytes, T lymphocytes and PMNs) distantly from other immune cells.

### An MS database as a tool for cell identification

**Figure 7:**
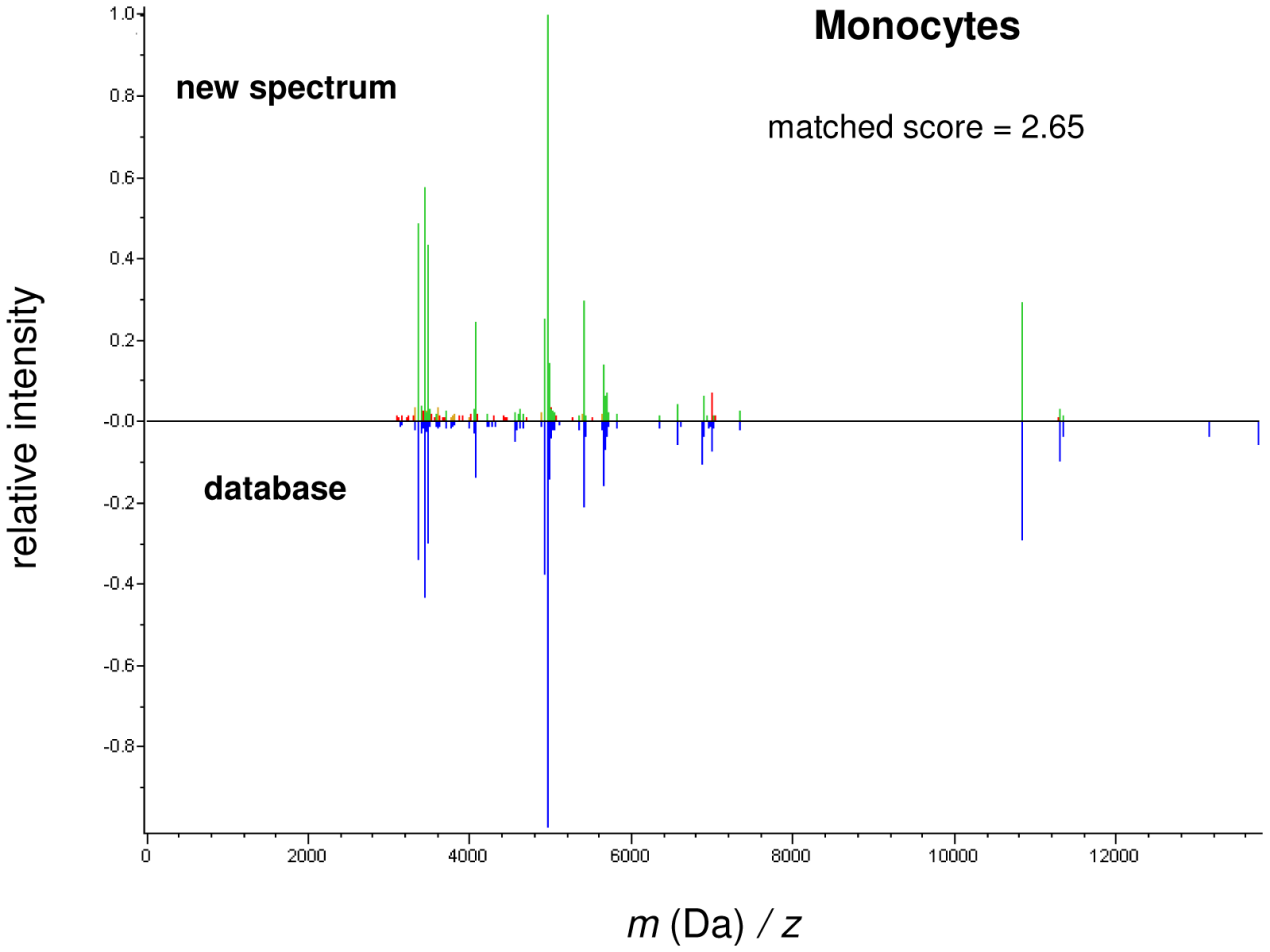
Efficiency of the database. A MALDI-TOF MS spectrum of unknown cells (here, monocytes from a blood donor) was compared to the averaged spectrum of monocytes generated from the database using the BioTyper software. The score indicates the identification of the tested cell population as monocytes.

We tested the efficiency of the database in three different ways. First, we compared a new monocyte sample with the mean spectrum of monocytes generated within the database (Fig. 7). The resulting score of 2.65 was highly significant and authenticated the tested cell population as monocytes. Second, we determined whether it is possible to identify cell populations within a cell mixture. Circulating monocytes and T lymphocytes (10^6^ each cell type) were mixed and the spectra obtained were compared to the database. Monocytes and T lymphocytes were respectively identified with a correct score of 2.25 for both monocytes and T lymphocytes. Third, the MALDI-TOF MS profile of PBMCs was analyzed according to the same procedure. Although the proportion of monocytes was small compared to T lymphocytes (about 1/7), we identified both T lymphocytes (with a score of 2.22) and monocytes (with a score of 2.21). Taken together, these results demonstrate that we were able to identify monocytes and T lymphocytes in a complex mixture, and they suggest that MALDI-TOF MS allows the confident identification of cell subsets in tissues.

## Discussion

In this report, we showed that a MALDI-TOF MS approach was able to identify intact immune cells. This method was rapid and easy to perform and did not require any additional components (such as specific antibodies), in contrast to flow cytometry. In addition, the repertoire of analyzed molecules is different between MALDI-TOF MS and flow cytometry because MALDI-TOF MS is applicable to soluble molecules with a molecular weight ranging from 2 to 20 kDa, whereas flow cytometry detects surface markers or intracellular proteins through permeabilization procedures. Our MALDI-TOF MS approach extended to eukaryotic cells an approach previously used for bacterial identification [12]–[17], [28]. In this study, intact primary or cultured cells were washed in saline to eliminate contamination by components such as cytokines or albumin contained in FCS, and thawed samples were deposited on the MALDI target in which HCCA matrix was added. Clearly, spectra were constituted by a collection of peaks, and their masses and relative intensities varied according cell origin. Other attempts have been performed to analyze MALDI-TOF MS profiles of whole eukaryotic cells. A recent report shows that MALDI-TOF MS typing is efficient to characterize 66 cell culture samples representing 34 species from insects to primates. Spectra of each cell type were composed of a variety of peaks with different masses and intensities, demonstrating the feasibility of our approach. However, as cell samples are treated by ethanol and formic acid/acetonitrile before assay [19], the two methods are not superimposable. Another report describes MALDI-TOF MS spectra from K562, BHK21 and GM15226 cell lines after lysis in 2,5-dihydroxybenzoic acid matrix solution. Again, obtained spectra show common peaks among the three cell types and specific peaks [18], demonstrating that MALDI-TOF MS performed on crude extracts of mammalian cell types may be useful to easily identify different cell types. In addition, we demonstrated that freezing cells at −80°C was sufficient to obtain high quality spectra, whereas cell lysis increased background noise in a non-interpretable way. Cell sonication led to spectra that were non-reproducible and peaks that were not entirely superimposable with those found in viable and frozen cells. The cell concentrations used to obtain significant spectra were relatively high, but were still less than those required for transcriptomics, another type of global method. In a prior study employing MALDI-TOF MS to analyze human macrophages, the extraction of membrane proteins requires specific protocols and a large quantity of cells [4].

We demonstrated that the spectra of immune cells were specific since they were markedly distinct from those of unrelated cell lines and differed between related immune cells. This specificity was supported by a set of peaks that represent the MS signature of each cell type. In addition, this study enabled us to develop a cell database comprised of 22 cell types representing diverse lineages of eukaryotic cells [29]. The database relies on the creation of a specific reference spectrum for each cell type and a score that validates the identification. These data have two major applications: first the establishment of a dendrogram of eukaryotic cells, and second, the analysis of mixed cell populations. The dendrogram revealed two major branches: one cluster of insect cells, amoebas and RBCs, and another cluster with immune cells and cell lines. Among the circulating leukocytes, the distance was smaller between monocytes and T lymphocytes, which are functionally distinct, than between monocytes and PMNs, although both are phagocytic cells. Monocytes, T lymphocytes and PMNs were in branches distinct from tissue immune cells such as macrophages and DCs. The divergence between monocytes and MDMs is consistent with previous transcriptomic studies in which each cell type had a specific program [30]. In addition, maturation from monocytes is a common feature of MDMs and DCs, and this accounts for the clustering of these two cell types. MDMs and DCs remained markedly distant in the dendrogram, which underlines their functional divergence. Interestingly, the clustering between cell types seemed independent of species origin. Indeed, human MDMs and murine BMDMs were close in the dendrogram. Similarly, the distance between the human THP-1, murine J774 and canine DH82 monocytic cells was low. We found that the position of primary trophoblasts was surprising: close to macrophage cell lines and distant from trophoblast cell lines (JEG and BeWo cells).

The use of the database enabled us to identify different cell populations among cell mixtures. The common signature of monocytes among individual donors was robust. Additionally, monocytes and T lymphocytes were accurately identified when they were mixed. Furthermore, the specific signatures of monocytes and T lymphocytes were found when PBMCs were studied. We suggest that the MALDI-TOF MS approach can be used to identify different cell types among tissue infiltrates. In addition, it is likely that its discriminative power is similar to that of genomic [29], proteomic and transcriptomic approaches [30]. Recent proteomic and transcriptomic approaches allow the discrimination between CD14^+^CD16^−^ and CD14^+^CD16^+^ monocyte subsets. Similarly, we preliminarily found that the activation of monocytes and macrophages resulted in specific spectra that correlated with their transcriptomic patterns (manuscript in preparation).

In conclusion, we developed a new method for identifying immune cells based on a MALDI-TOF MS approach. A major advantage of this method compared to the usual techniques is the lack of purification steps and staining procedures, which often lead to cell activation. The cell database we constructed was useful for identifying a cell type within a cell mixture, and it could potentially be used to identify different functional states of a cell population such as monocytes or macrophages.

## Supporting Information

Figure S1 MALDI-TOF MS spectra of monocyte preparations. Human monocytes (106 cells per assay) were collected in 10 µl of PBS, and 1 µl was deposited on the MALDI target. Representative MALDI-TOF MS spectra are shown: A, in the absence of monocytes; B, lysed monocytes; C, sonicated monocytes.
(0.30 MB TIF)
