# Supplementary figures and images for: Global Analysis of Circulating Immune Cells by Matrix-Assisted Laser Desorption Ionization Time-of-Flight Mass Spectrometry
Source: PLoS One. 2010 Oct 27;5(10):e13691. doi: 10.1371/journal.pone.0013691 (PMC2965159; doi:10.1371/journal.pone.0013691)

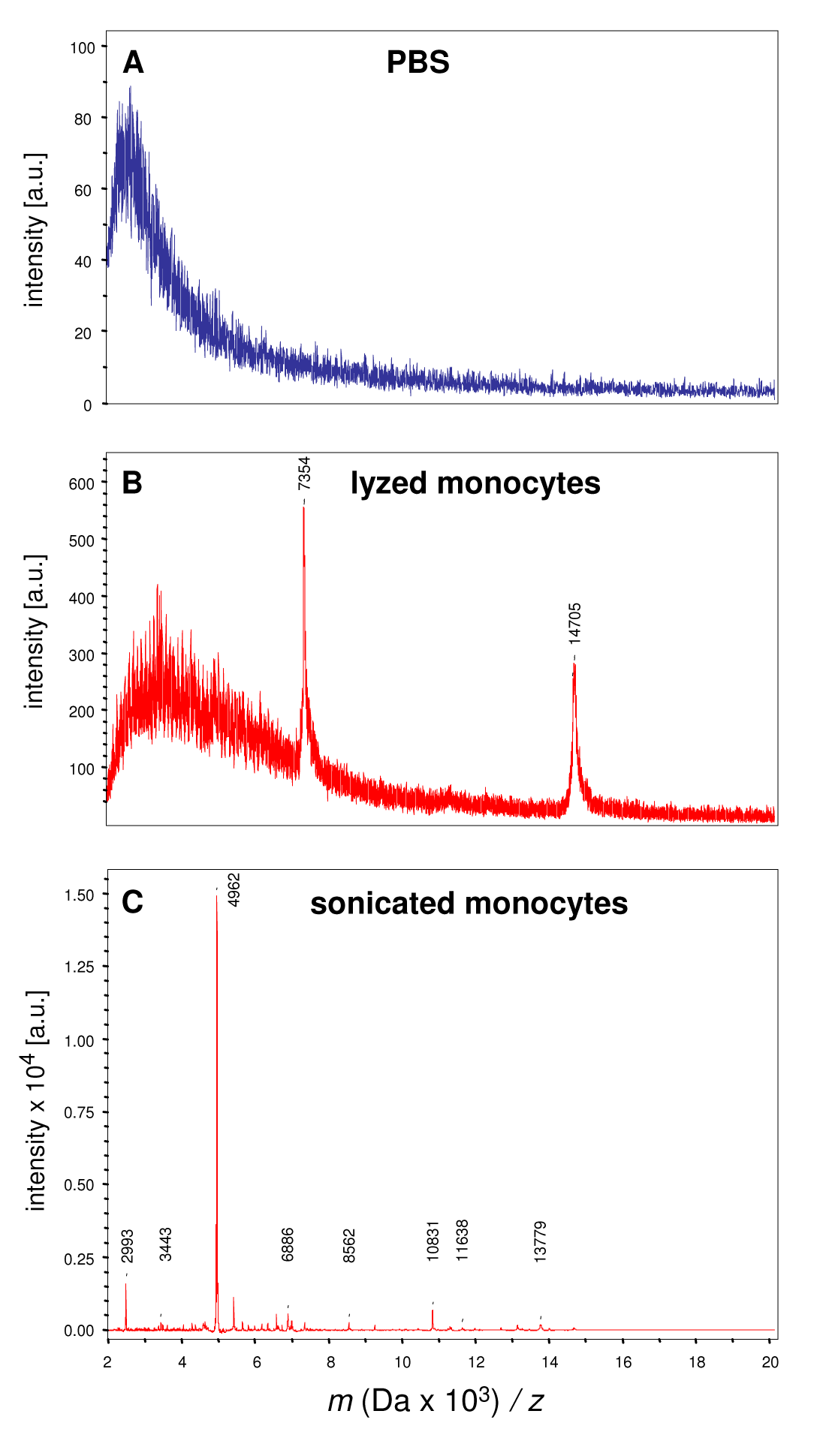

Supplement: Figure S1 — MALDI-TOF MS spectra of monocyte preparations. Human monocytes (106 cells per assay) were collected in 10 µl of PBS, and 1 µl was deposited on the MALDI target. Representative MALDI-TOF MS spectra are shown: A, in the absence of monocytes; B, lysed monocytes; C, sonicated monocytes. (0.30 MB TIF) [file pone.0013691.s001.tif]
